# Tone and vowel perception delay: long-term effects of late cochlear implant in children with prelingual deafness

**DOI:** 10.3389/fnhum.2025.1516931

**Published:** 2025-03-11

**Authors:** Maojin Liang, Peng Peng, Jiahao Liu, Zhengye Wang, Kaiying Lai, Junbo Wang, Yiqing Zheng, Suiping Wang

**Affiliations:** ^1^Department of Otolaryngology, Sun Yat-sen Memorial Hospital, Institute of Hearing and Speech-Language Sciences, Sun Yat-sen University, Guangzhou, China; ^2^School of Psychology, South China Normal University, Guangzhou, China; ^3^Philosophy and Social Science Laboratory of Reading and Development in Children and Adolescents (South China Normal University), Ministry of Education, Guangzhou, China; ^4^Research Center for Brain Cognition and Human Development, Guangzhou, Guangdong, China; ^5^Guangzhou Institute of Educational Research, Guangzhou, China

**Keywords:** cochlear implant, speech perception, sensitive period, electroencephalography, event-related potential, mismatch negativity, late discriminate negativity

## Abstract

**Purpose:**

The influence of the duration of the subsequent rehabilitation period on the perception of Mandarin tones and vowels has not been fully investigated. This study explores phoneme perception and event-related potential (ERP) responses in prelingually cochlear implant (CI) children, comparing early (eCI) vs. late implantation (lCI) with 5-year rehabilitation.

**Method and results:**

This study involved 19 early cochlear implanted (eCI) children, 19 late cochlear implanted (lCI) children (both right-ear implantation), and 21 normal-hearing (NH) children as a control group. EEG data were recorded for all groups during a passive multi-feature auditory oddball paradigm, involving deviant and standard stimuli. Behavioral performance was also assessed to validate Electroencephalogram-based (EEG-based) indicators. Results showed that the lCI group had significantly longer P2 latency and amplitude in the ERP test compared to the NH group, but not the eCI group. Both CI groups had smaller mismatch negativity (MMN) amplitudes than the NH group in tone and consonant conditions. The lCI group showed larger late discriminative negativity (LDN) amplitudes than the eCI group in tone and vowel conditions. Behavioral results aligned with EEG findings, with the eCI group performing better than the lCI group in tone and vowel conditions. The LDN amplitude in CI groups is larger for both tone and vowel conditions when the age at cochlear implantation is older.

**Conclusion:**

These results indicate that (1) the earlier the age of implantation, the better the ability to perceive tones; (2) Implantation age of CI showed no significant effect on consonant perception; (3) The LDN component may be an indicator to discriminate eCI and lCI children in terms of Mandarin tone and vowel perception. (4) The P2 latency and amplitude may be an indicator to discriminate NH and CI children in phoneme perception.

## 1 Introduction

Cochlear implants (CIs) have provided positive outcomes for individuals with profound to severe hearing loss (Kral and Eggermont, 2007), but there are some children for whom speech rehabilitation has not been satisfactory, particularly those with late implantation, who tend to have poor outcomes (Sharma et al., 2015). This may be related to the sensitive period for phoneme perception.

Auditory deprivation can lead to abnormal development of the central auditory system, especially when the auditory deprivation occurs during the sensitive period from birth to 3.5 years (Sharma et al., 2002, 2009; Sharma and Dorman, 2006). As auditory deprivation may limit the speech development in deaf children, it is reasonable to assume that the CI surgery performed during the sensitive period may promote the development of speech perception skills (Kral et al., 2006, 2019). Previous EEG/ERP studies have shown that deaf children who receive CI before the age of 3.5 years (early implantation) have the best post-operative speech development, whereas those who receive CI after the age of 3.5 years (late implantation) have poor speech rehabilitation (Sharma et al., 2002). There are plenty of researches in people with normal hearing (NH), the density of synapses in the temporal lobe peaks around the age of 3.5 years and then starts to decrease (Huttenlocher and Dabholkar, 1997; Sharma et al., 2015). Therefore, setting the cutoff age for implantation at 3.5 years old might be rational for the present study.

Notably, studies have found that different Chinese phonemes have different critical periods for speech acquisition (Chen et al., 2017). Therefore, in order to investigate the Chinese phoneme perception ability of CI children, it is necessary to consider not only the critical period of auditory system development, but also the critical period of phoneme processing.

As a tonal language, the difference between Chinese and other non-tonal languages lies in its tonal characteristics (Shen and Froud, 2019). In Chinese, tone not only has the variation of pitch contour, but also contains rich semantic information. For example, if the syllable ‘ma’ is in the first tone, it means ‘mother’. Hua and Dodd (2000) found that NH children acquired tones by the age of 2, vowels by around 3.5 and consonants by around 5. Based on the phonological saliency hypothesis, tones and vowels are indispensable elemental units, and the acquisition of vowels and tones is earlier than that of consonants in Mandarin monolingual learners. This may be due to the fact that the acquisition of Mandarin Chinese phonological features requires a certain degree of cortical maturation, and the timing of cortical maturation and the amount of training required vary for different elemental phonetic units (Wewalaarachchi et al., 2017; Wu et al., 2007). According to the order of Chinese phoneme acquisition, if the deaf children receive CI earlier, the more opportunities they may have to acquire tone and vowel perception. Despite the limitation of sound transmission (i.e., spectra) via CI dependent on intracochlear device placements and interindividual differences. Therefore, we wondered if there were differences in tone and vowel processing between early and late CI children.

Considering the critical period of Chinese phoneme acquisition, the rehabilitation period is a very important factor in the phoneme perception of children with CI. However, previous studies on Mandarin phoneme perception have ignored the factor of rehabilitation period. For example, the best age for consonant acquisition was about 5 years old. Some studies selected CI children with a long recovery period (about 5 years after surgery), and found that their recognition ability was worse than that of NH children, and that consonant perception ability was negatively correlated with the implantation age (Liu et al., 2013); others selected CI children with a rehabilitation period of about 2 years, and found that consonant perception was not related to the implantation age (Wu and Yang, 2003). The differences in these results may also be related to the length of rehabilitation duration in different studies. CI children receive auditory and verbal information much later than normal hearing children, and may therefore acquire different basic phoneme units later, which means that they may be delayed at the end of the sensitive period of language acquisition. However, most studies have only investigated speech perception after short-term rehabilitation (i.e., less than 2 years after CI surgery), and CI children may not have acquired all the phonological features by the age at which normal hearing children acquire these features. Therefore, CI children with a longer duration of rehabilitation (e.g., around 5 years) are selected to ensure that they have sufficient sensitive periods for the acquisition of tones, vowels and consonants acquisition to better explore the effect of implantation age on Mandarin elemental units’ perception.

In the present study, behavioral and neurophysiological tasks were used to investigate the phonemes processing in Chinese syllables in prelingually deaf CI children who have been rehabilitated for approximately 5 years. We used EEG techniques combined with the multi-feature oddball paradigm (Näätänen et al., 2004) to compare neural responses to elemental phonemes units (tones, vowels and consonants) in children with early and late CI after around 5-years rehabilitation duration. Previous studies have focused on the components associated with auditory perception, including P2 (Lee et al., 2011; Tremblay et al., 2014; Zhang et al., 2011) and mismatch negative (MMN) (Sharma et al., 2002). Among the various components, the P2 latency has been implicated in poor speech responses (Zhang et al., 2011). Furthermore, the P2 amplitude has been reported to exhibit an association with response time (Lee et al., 2011; Tong et al., 2009) and learning processing of phonemes (Tong et al., 2009; Tremblay et al., 2014). Despite these findings, the functional role of the P2 component remains poorly understood, particularly with regard to its ability to differentiate performance between normal-hearing (NH) and cochlear implant (CI) groups. Nonetheless, it has been documented that the P2 component is correlated with neural adaptation (Zhang et al., 2009) and object representation (Tremblay et al., 2014) in speech perception.

An increased MMN amplitudes have been associated with good speech outcomes in children with CI (Zhang et al., 2011). The late discriminative negativity (LDN) reflects additional processing of auditory stimuli when standard and deviant stimuli are indistinguishable (Bishop et al., 2010; Čeponiene et al., 1998; Shestakova et al., 2003; Uhlén et al., 2017). Studies have found that the LDN component is related to children’s language ability and cognitive behavioral performance, and is associated with more advanced neurocognitive processing (Kuuluvainen et al., 2016). Furthermore, LDN occurs in young children, but not in adults (Liu et al., 2014; Putkinen et al., 2012). These studies suggest that the LDN component may be an important indicator of auditory system processing.

However, when investigating the neural correlates between ERP components and speech performance, some studies have focused exclusively on a limited number of these components and acoustic characteristics. For instance, (Tremblay et al., 2014) examined the P2 component using the canonical oddball paradigm (Lee et al., 2011). Conversely, other studies have analyzed both the latencies and amplitudes of the P2 and MMN components, albeit solely within the context of speech syllables using the canonical oddball paradigm (Hu et al., 2021; Zhang et al., 2011). Consequently, we investigated the relationship between phoneme perception and the characteristic of these ERP components, including latency and amplitude, which focused on the P2, MMN and LDN components. In contrast to the aforementioned studies, the current research employed a passive auditory multi-feature oddball paradigm known as Optimum-2 (Näätänen et al., 2004). This paradigm was utilized to present four fundamental phonemes: tone, vowel, consonant, and syllable. This paradigm was considered superior to the canonical one, since participants were able to rapidly capture a broader range of acoustic characteristics as mentioned (Näätänen et al., 2004). The processes of early acoustic encoding and later cognitive processing were revealed by the MMN and LDN components, respectively. We hypothesized that the MMN would be smaller and the LDN would be larger in CI children with late cochlear implantation than in CI children with early cochlear implantation, indicating a reduced capacity for sensory and strong top-down cognitive processing in lCI children. Given that numerous reports have associated the P2 component with neural adaptation and training in response to auditory stimuli in the cerebral cortex (Lee et al., 2011; Tong et al., 2009; Zhang et al., 2011), where better performance by participants is correlated with shorter latencies and lower amplitudes; hence, we hypothesize that the P2 latency in the NH group will be shorter than that in the CI groups, and the amplitude will also be smaller in the NH group compared to the CI groups.

## 2 Materials and methods

### 2.1 Participants

Thirty-eight bilateral prelingual deaf CI children speaking Mandarin were recruited. All had a right-sided implant and used the same brand of implant (MED-EL). Among them, 19 children who received implants earlier than 3.5 years of age were divided into the early CI group (10 males), while the other 19 who received implants later than 3.5 years of age were included as the late CI group (9 males). Another 21 normal hearing Mandarin-speaking children were included as the NH group (9 males). The demographic information of the children in all three groups is summarized in Table 1. No significant difference was found between the early CI children and late CI children in the duration of rehabilitation [*F*_(1,36)_ = 2.402, *p* = 0.130, *η*_p_^2^ = 0.063]. The Raven’s Standard Progressive Matrices Test was used to measure the non-verbal IQ scores of children (Raven et al., 1998). There was a main effect of three groups on non-verbal IQ scores [*F*_(2,56)_ = 4.174, *p* = 0.020, *η*_p_^2^ = 0.130], with both CI groups scoring lower than the NH group [eCI *p* = 0.048; lCI: *p* = 0.049]. In addition, a marginally significant difference in age was found among the three groups [*F*_(2, 56)_ = 3.023, *p* = 0.057, *η*_p_^2^ = 0.097], and the lCI group had higher age than the eCI group [*p* = 0.094]. Therefore, age and nonverbal IQ were used as the covariates in further analyses. Ethical approval was obtained from the Human Research Ethics Committee for Non-Clinical Faculties of South China Normal University (No. 158). Written parental informed consent was obtained for each participant. A visual diagram of time windows, components and factors analyzed in the present study was illustrated in Table 2.

**Table 1:** Descriptive characteristics of NH and CI participants.

	NH group				eCI group				lCI group				*F*	*p*
	*n*	*M*	*SD*	Range	*n*	*M*	*SD*	Range	*n*	*M*	*SD*	Range		
AOI	/	/	/	/	19	1.9	0.8	1.0–3.0	19	4.0	0.4	3.7–5.0	*F*_(1,36)_ = 106.20	<0.001
DOR	/	/	/	/	19	6.2	1.8	4.4–10.5	19	5.5	1.2	3.2–7.3	*F*_(1,36)_ = 2.402	0.130
Age	21	9.3	1.6	6–12	19	8.2	2.1	5.8–12.5	19	9.4	1.5	6.5–12	*F*_(2,56)_ = 3.023	0.057
Nonverbal IQ	21	116	8.7	101–130	19	105	18	67–123	19	105	15	76–131	*F*_(2,56)_ = 4.174	0.020

NH group indicates normal hearing group; eCI group, early cochlear implant group; lCI group, late cochlear implant group; AOI, age of implantation; DOR, duration of rehabilitation.

**Table 2:** Visual diagram of time windows, components and factors analyzed in the present study.

Time window	ERP component	Factors	
		Latency	Amplitude
100-250 ms	P2	√	√
100-250 ms	MMN	√	√
300-500 ms	LDN	√	√

Undeniably, the auditory system maturation levels exhibit considerable variation following a 5-year rehabilitation period. Nevertheless, obtaining data from individuals who underwent cochlear implantation (CI) at the exact same age presents significant challenges. Consequently, to ensure a reliable factor in our manuscript, we have gathered data from a broad age range of children, spanning from 5.8 to 12.5 years old, as detailed in Table 1.

### 2.2 Stimuli

Each participant completed speech behavior assessments and auditory ERP tests. All sound materials were prepared with reference to the *Auditory Function Evaluation Standards and Methods* (Sun et al., 2007). The behavioral assessments included tone, vowel and consonant perception conditions. The tonal discrimination task included 48 pairs of the same stimuli and 32 pairs of different stimuli. Vowel discrimination task included 24 pairs of the same stimuli and 36 pairs of different stimuli. Consonant discrimination task included 40 pairs of the same stimuli and 60 pairs of different stimuli.

In the ERP tests, the standard stimuli were /da1/ (presented in Pinyin alphabet), while the deviant stimuli were /da4/, /ba1/, /du1/ and /bu4/. The four deviant stimuli differed from the standard stimuli in tone, consonant, vowel and syllable, respectively. The speech stimuli were recorded by an adult native male Mandarin speaker using the Neundo 4 software (Steinberg Media Technologies, Germany) at a sampling rate of 44.1 kHz and a resolution of 16 bits. All speech stimuli were digitally normalized to a set level of 75 dB SPL and a duration of 300 ms using Praat software (Boersma and Van Heuven, 2001), and the Sound Forge software (Boersma and Van Heuven, 2001; Boersma and Weenink, 2014) was used to normalize the fundamental frequency, intensity and duration of all sounds.

In this study, standard stimuli and deviant stimuli were selected according to the following criteria. (1) All speech stimuli had corresponding real Chinese characters (Lee et al., 2012). (2) the syllables /ba/, /da/, /du/, and /bu./ were frequently used in previous studies (Feng et al., 2019; Lee et al., 2012). (3) In the vowel condition, the standard stimulus /da1/ and the deviant stimuli /du1/ were identical in syllable but different in vowel. Based on Mandarin vowels classification (Cheng, 1966), /a/ is a low vowel and /u/ is a high vowel. Moreover, /a/ is front vowel and /u/ is a back vowel. Therefore, we assigned /da/ as the common standard and /du/ as the deviant. (4) In the consonant condition, /b/ is a bilabial consonant, /d/ is an apical consonant, two consonants belong to different categories. (5) In the tone conditions, we selected the high level tone (T1) as the standard stimulus and the high falling tone (T4) as the deviant stimulus, because previous studies have found that when T1 was the standard stimuli, T4 as the deviant stimuli induced the largest MMN compared to the high rising tone (T2) or the low dipping tone (T3) as the deviant stimulus (Yang et al., 2008).

### 2.3 Procedure

The children underwent EEG testing while listening to a passive multi-oddball auditory task. Speech stimuli were presented in a sequence of a standard stimulus followed by a deviant one, and the sequence was pseudo-random to avoid two consecutive deviant stimuli being identical. A 128-channel dense array EEG system (EGI, USA) was used to collect EEG data. The participating children were seated in a comfortable chair adjusted to the appropriate height, and were instructed to watch a muted, self-selected animated film without paying attention to the sound stimuli from the loudspeaker. Speech stimuli were presented at a hearing level of 75 dB SPL at an angle of 45 degrees to the left and to the right ear through two loudspeakers placed 80 cm in front of the child (Ni et al., 2021).

In the present study, the passive auditory multi-feature oddball paradigm (Optimum-2) (Näätänen et al., 2004) was used, in which the four types of deviant stimuli were presented in the same order, with three standards between each two successive deviant stimuli, i.e., the same standard stimuli alternated with the different types of deviant stimuli. Compared to the optimum-1 condition proposed by Näätänen et al. (2004), the optimum-2 paradigm produced smaller MMN amplitudes, but the overall results were similar to those obtained with the traditional oddball paradigm (Näätänen et al., 2004). Thus, the paradigm used in the present study was able to record neural responses to multiple deviant stimuli in a relatively short EEG recording time, allowing for a more comprehensive assessment of the ability of CI children to recognize the elemental phonetic unit. Specifically, the total number of trials in the experiment was 1,120, including 140 trials for each type of deviant stimulus, and 560 trials for the total number of standard stimuli. The experiment was preceded with 10 trials of standard stimuli. The inter-stimulus interval (ISI) was randomized between 650 and 750 ms. The duration of the EEG recording was approximately 20 min. The EEG was recorded with an EGI Net Amp 300 (EGI, USA) at a sampling rate of 1,000 Hz. Consisted with other researches (Kuntzelman and Miskovic, 2017; Liang et al., 2014; Lui et al., 2021), the impedance was kept below 40 kΩ for all channels during the experiment using this EEG collection device.

In addition to the EEG experiment, each participant also completed the speech discrimination tests for tones, vowels and consonants, using a same-different paradigm. Specifically, on each trial the children first heard a sound for 500 ms, then a blank screen appeared on the screen for 300 ms, and finally another sound stimulus was presented for 500 ms. Participants were asked to judge as quickly as possible whether two consecutive sounds were the same or different. In the practice sessions, participants received feedback after each trial to ensure that all participants understood the experimental requirements. In the formal experiment, no feedback was given after each trial. The total duration of this behavioral experiment was approximately 30 min.

### 2.4 Data analysis

The EEG data were pre-processed using EEGLAB Toolbox and analyzed using ERPLAB Toolbox (Delorme and Makeig, 2004) in MATLAB with custom code for the following steps. First, the EEG signals were filtered with a 0.5–30 Hz band-pass, and then resample to 500 Hz. Independent component analysis (ICA) and principal component analysis (PCA) were then applied, and components attributed to eye movements, eye blinks and CI artifacts were removed using SASICA Toolbox (Ni et al., 2021). The data was manually checked to ensure that the artefacts caused by unexpected movement of the participants were eliminated. After data pre-processing, the eye blinks and CI artifacts were removed, then the epoch was set at 100 ms pre-stimulus and 700 ms post stimulus, and epochs with extreme amplitudes (exceeding ±100 μV) were rejected. Valid epochs were then averaged for the standard and deviant stimuli in each condition. All responses at each electrode site were re-referenced using REST Toolbox. A fronto-central region (E5, E 6, E7, E12, E13, E106 and E112 in an EGI 128-channel HGS net) was selected for further analysis, because the MMN and LDN responses usually occurred with a fronto-central distribution (Chen et al., 2018), in line with previous auditory MMN findings (Uhlén et al., 2017; Näätänen et al., 2007).

In the present study, the P2 (P200) was determined as the positive peak at 100–250 ms (Lee et al., 2011; Tremblay et al., 2014; Zhang et al., 2011), P2 peaks of each epoch were detected and combined for latency and amplitude analysis. In each condition, after subtracting the neural responses to the deviant stimuli from those to the standard stimuli, and combined with previous studies, the MMN and LDN were identified as the most negative peak within 100–250 ms and 300–500 ms, respectively (Bishop et al., 2011; Sharma et al., 1997). The MMN and LDN amplitudes were obtained by calculating the mean voltage of samples with a 40-ms time span around the peak latency.

SPSS 25.0 software (IBM, USA) was used for the statistical analysis. The significance level was set at *α* = 0.05, unless otherwise stated. To examine differences in behavioral results and ERP responses among the three groups, a two-way repeated measures analysis of variance (ANOVA) was used with groups (NH, eCI and lCI) as the between-subject factor, and the conditions as the within-subject factor. We conducted individual Shapiro–Wilk tests for both amplitudes and latencies for each subject group. The non-normally distributed data was analyzed with non-parametric repeated measures analysis of variance (ANOVA) using the Aligned Rank Transform Tool (ARTool) (Wobbrock et al., 2011; Elkin et al., 2021). Age and non-verbal IQ scores were covariates for statistical analysis. If the sphericity assumption about the variance of differences was violated, the Greenhouse–Geisser correction was used to compensate for violations of sphericity. Correction for multiple comparisons was applied where appropriate.

## 3 Results

### 3.1 Behavioral results

**Figure 1:**
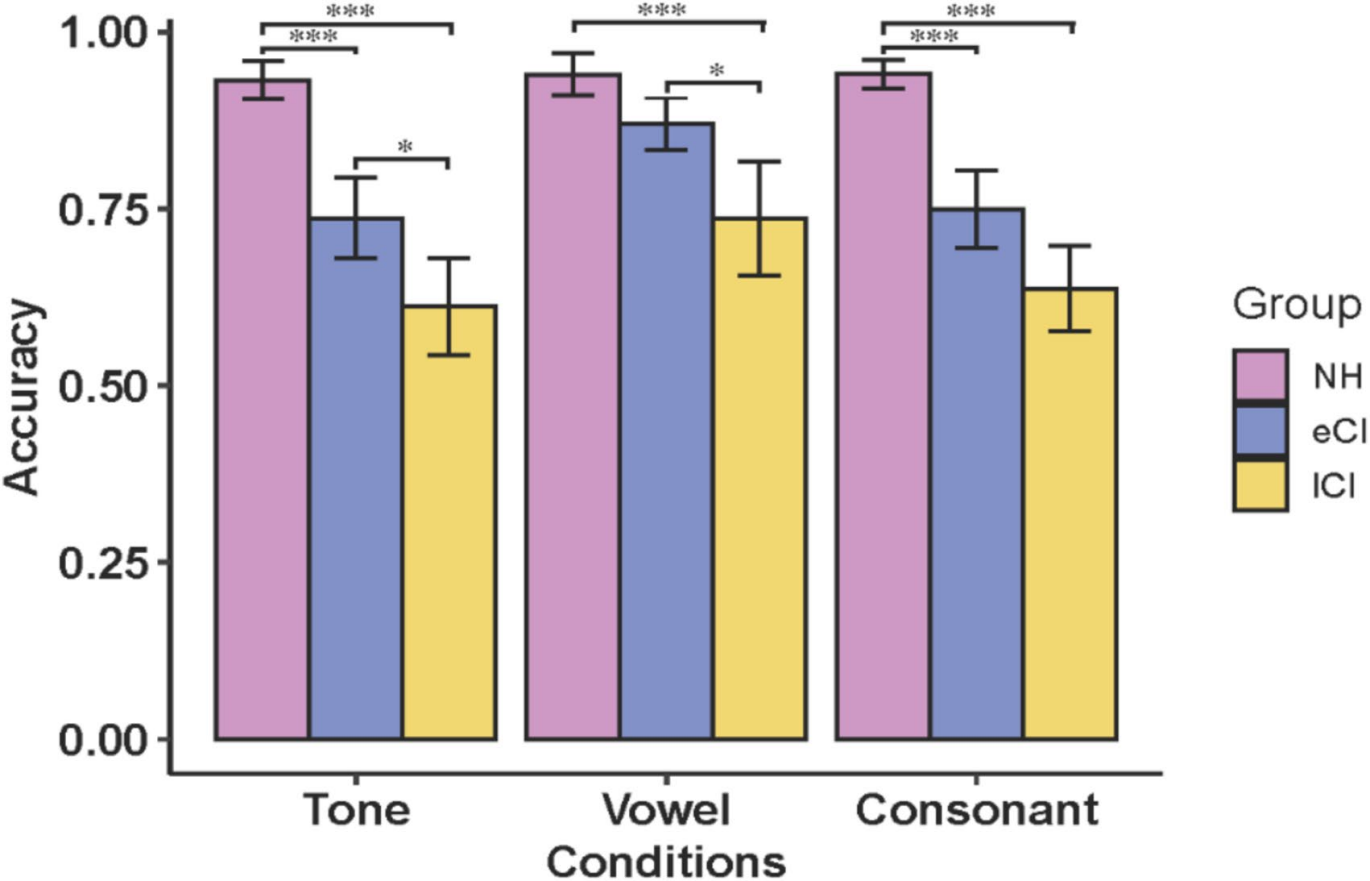
Accuracy of NH, eCI and lCI on the tone, vowel and consonant tests. NH: children with normal hearing; eCI: children with early cochlear implantation; lCI: children with late cochlear implantation. ***p < 0.001, *p < 0.05. Error bars: standard error of the mean across subjects.

Figure 1 shows a summary of the results of the three tasks for the NH, eCI and lCI groups. ANOVA was carried out with group (NH, eCI and lCI) as the between-subject factor, and condition (tone, vowel and consonant) as the within-subject factor. There was a significant interaction between group and condition [*F*_(4, 106)_ = 3.318, *p* = 0.013, η_p_^2^ = 0.111]. Post-hoc analyses revealed that the accuracy of tone identification was significantly higher for NH children than for eCI children [*t* = 4.645, *p* < 0.001] and lCI children [*t* = 8.207, *p* < 0.001], and the accuracy of eCI children was significantly higher than that of lCI children [*t* = 3.339, *p* = 0.041]. In the vowel condition, the accuracy of lCI children was significantly lower than that of eCI children [*t* = −3.307, *p* = 0.046] and NH children [*t* = −5.509, *p* < 0.001]. In the consonant condition, NH children performed significantly better than eCI children [*t* = 5.174, *p* < 0.001] and lCI children [*t* = 8.144, *p* < 0.001], but no significant difference was found between eCI and lCI children [*t* = 2.739, *p* = 0.259]. The significant group effect was found [*F*_(2, 53)_ = 40.883, *p* < 0.001, *η_p_^2^* = 0.607], NH children performed better than eCI children [*t* = 4.913, *p* < 0.001] and lCI children [*t* = 9.032, *p* < 0.001], eCI children performed better than lCI children [*t* = 3.878, *p* < 0.001]. There was a marginally significant was found in condition factor [*F*_(2, 106)_ = 2.744, *p* = 0.069, *η_p_^2^* = 0.049], with accuracy in the vowel condition being significantly lower than in the tone condition [*t* = −5.922, *p* < 0.001] and higher than in the consonant condition [*t* = 4.899, *p* < 0.001]. However, no significant interaction between group and condition was found for reaction time [*F*_(3.315, 87.842)_ = 1.579, *p* = 0.196, *η_p_^2^* = 0.056], and no significant effect was found in condition factor [*F*_(1.657, 87.842)_ = 0.981, *p* = 0.365, *η_p_^2^* = 0.018]. We found a significant effect in the group factor [*F*_(2, 53)_ = 7.292, *p* = 0.002, *η_p_^2^* = 0.056], reaction time was longer in NH children than in lCI children [*t* = 3.819, *p* < 0.001].

### 3.2 Delayed P2 latency and larger amplitude for the phoneme perception in lCI children

**Figure 2:**
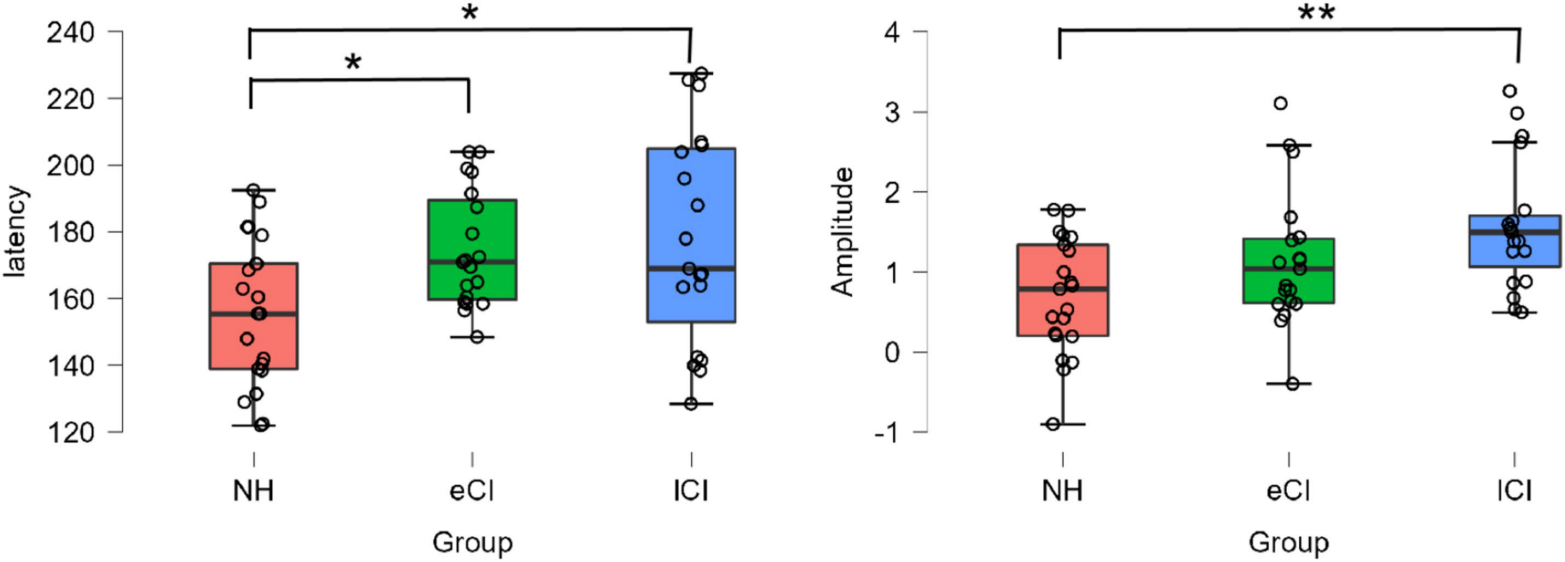
The P2 peak latencies (**left panel**, ms) and amplitudes (**right panel**, *μV*) of each group of NH, eCI and lCI. *p < 0.05 for one-star significance and **p < 0.01 for two-star significance.

**Figure 3:**
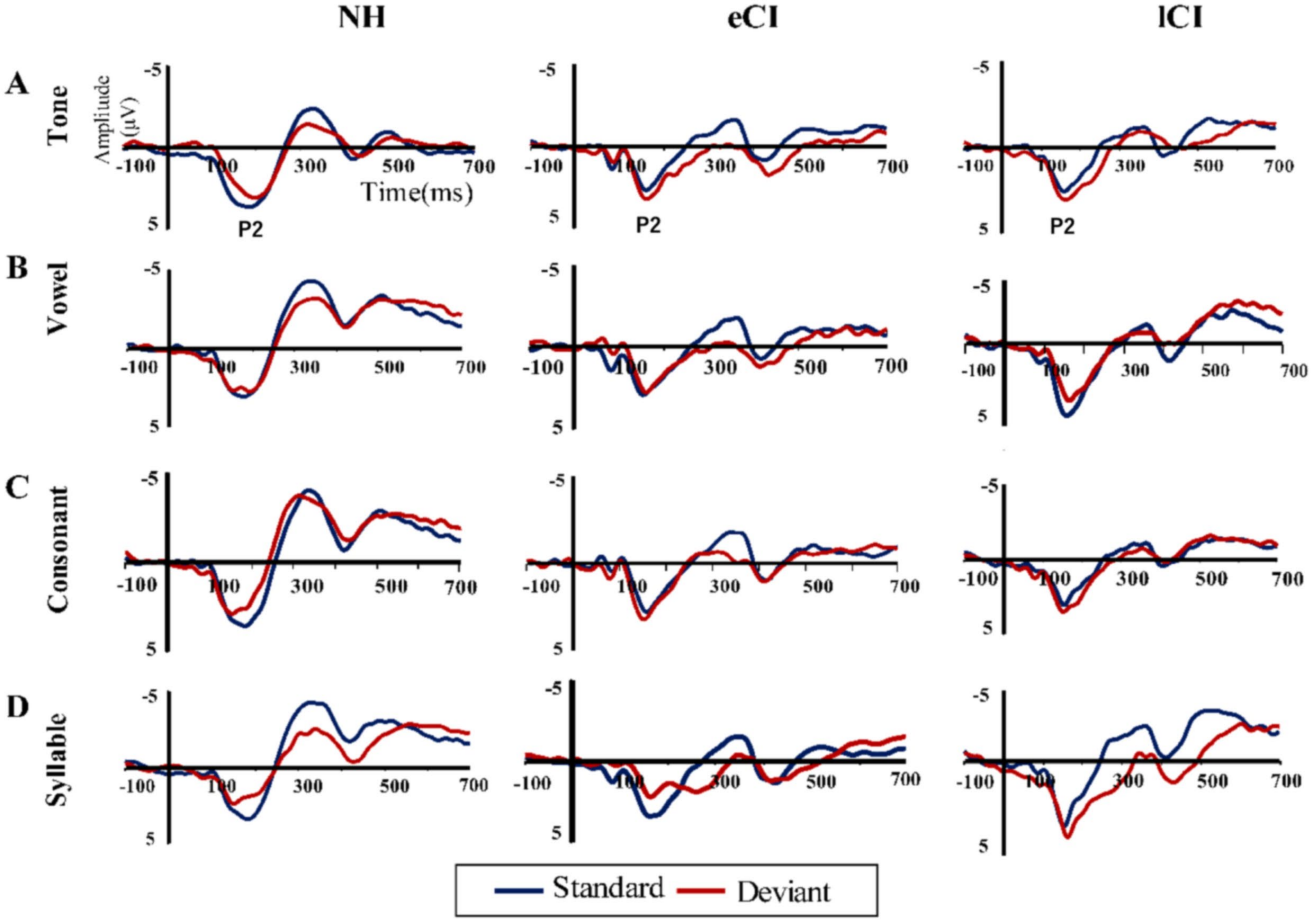
Grand average event-related potentials to standard stimuli (blue line) and those to the deviant stimuli (red line) at the front-central region in **(A)** tone, **(B)** vowel, **(C)** consonant, and **(D)** syllable conditions.

A significant effect of group was found for P2 peak latency [*F*_(2, 56)_ = 4.606, *p* = 0.014, *η_p_^2^* = 0.141] as shown in the left of Figure 2. The P2 latency of the lCI groups (177.79 ± 31.896 ms) was significantly longer than that of the NH group (156.21 ± 21.908 ms) [*t* = −2.787, *p* = 0.022], and between the eCI and NH groups [*t* = −2.382, *p* = 0.041]. But we failed to find a difference between the eCI (174.69 ± 17.598 ms) and lCI groups [*t* = −0.395, *p* = 0.695]. There was a significant effect of condition [*F*_(3,168)_ = 5.583, *p* = 0.001, *η*_p_^2^ = 0.091], but no significant effect was found in the interaction between condition and group [*F*_(3,168)_ = 0.406, *p* = 0.874, *η*_p_^2^ = 0.014] (P2 waveform is shown in Figure 3).

A significant effect of group was also found for P2 peak amplitude [*F*_(2, 56)_ = 6.095, *p* = 0.004, *η_p_^2^* = 0.179] as shown in the right of Figure 2. The P2 amplitude of the lCI groups (1.573 ± 0.800 *μV*) was significantly longer than that of the NH group (0.70 ± 0.727 *μV*) [*t* = −3.488, *p* = 0.003]. But we failed to find a difference between the eCI (1.15 ± 0.845 *μV*) and lCI groups [*t* = −1.649, *p* = 0.155] and between the eCI and NH groups [*t* = −1.798, *p* = 0.155]. However, there was no significant effect of condition [*F*_(3,168)_ = 0.765, *p* = 0.515, *η*_p_^2^ = 0.013] nor interaction between condition and group [*F*_(6,168)_ = 0.743, *p* = 0.616, *η*_p_^2^ = 0.026].

### 3.3 Smaller MMN amplitude for the tone and consonant conditions in CI children

**Figure 4:**
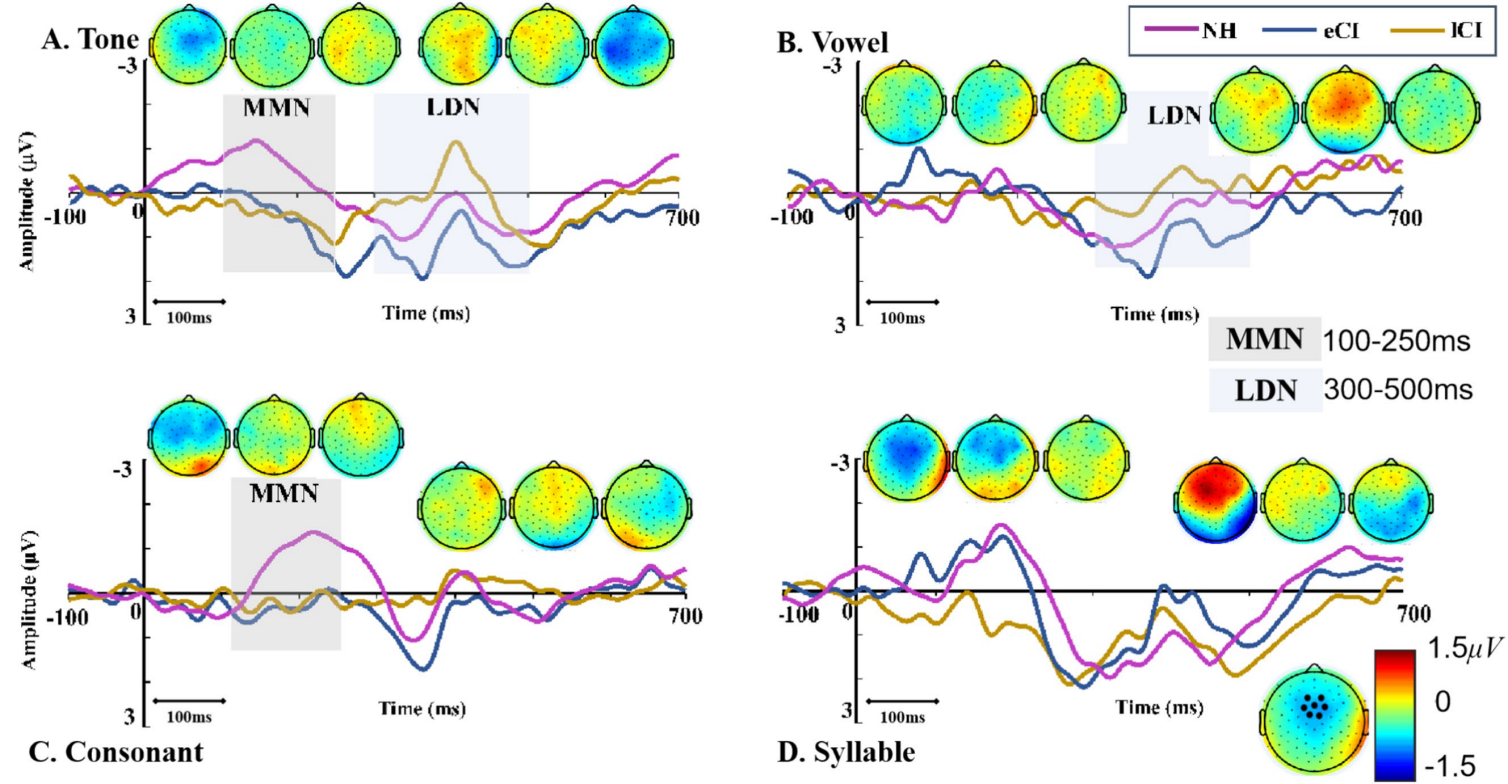
MMN and LDN of NH, eCI and lCI groups in four conditions: **(A)** Tone, **(B)** Vowel **(C)** Consonant **(D)** Syllable. Light gray and light blue vertical bars indicate significant difference in MMN and LDN. MMN: mismatch negativity, LDN: late discriminative negativity. Each triple-scalp topography groups are arranged from left to right with NH, eCI, and lCI in corresponding order. In each condition, the two sets of triple-scalp topography groups represent the scalp topographies of MMN and LDN, respectively.

The MMN waveforms were shown in Figure 4. The results showed a significant interaction between group and condition [*F*_(6,162)_ = 3.481, *p* = 0.003, *η*_p_^2^ = 0.114]. The results of further post-hoc tests showed that in the tone condition, MMN amplitudes were significantly larger in NH children than in the CI children [*p* = 0.005 for eCI children and *p* = 0.006 for lCI children], but no significant difference was found between the eCI group and the lCI group. Similar results were also found in the consonant condition, that is, NH children yielded significantly larger MMN amplitudes than that of the eCI children [*p* = 0.044] and the lCI children [*p* = 0.047], while no significant difference was found between the eCI and lCI children. However, in both the vowel and syllable conditions, no significant difference was observed in MMN amplitudes among the three groups [*p_s_* > 0.459]. Furthermore, there was a significant main effect of group [*F*_(2, 54)_ = 15.844, *p* < 0.001, *η*_p_^2^ = 0.370], the MMN amplitude was significantly larger in NH children than in eCI children [*p* < 0.001] and lCI children [*p* < 0.001], and no significant difference between eCI children and late CI children [*p* = 0.573]. However, there was no significant main effect of condition [*F*_(3, 162)_ = 1.576, *p* = 0.197, *η*_p_^2^ = 0.028] (Figure 4).

**Figure 5:**
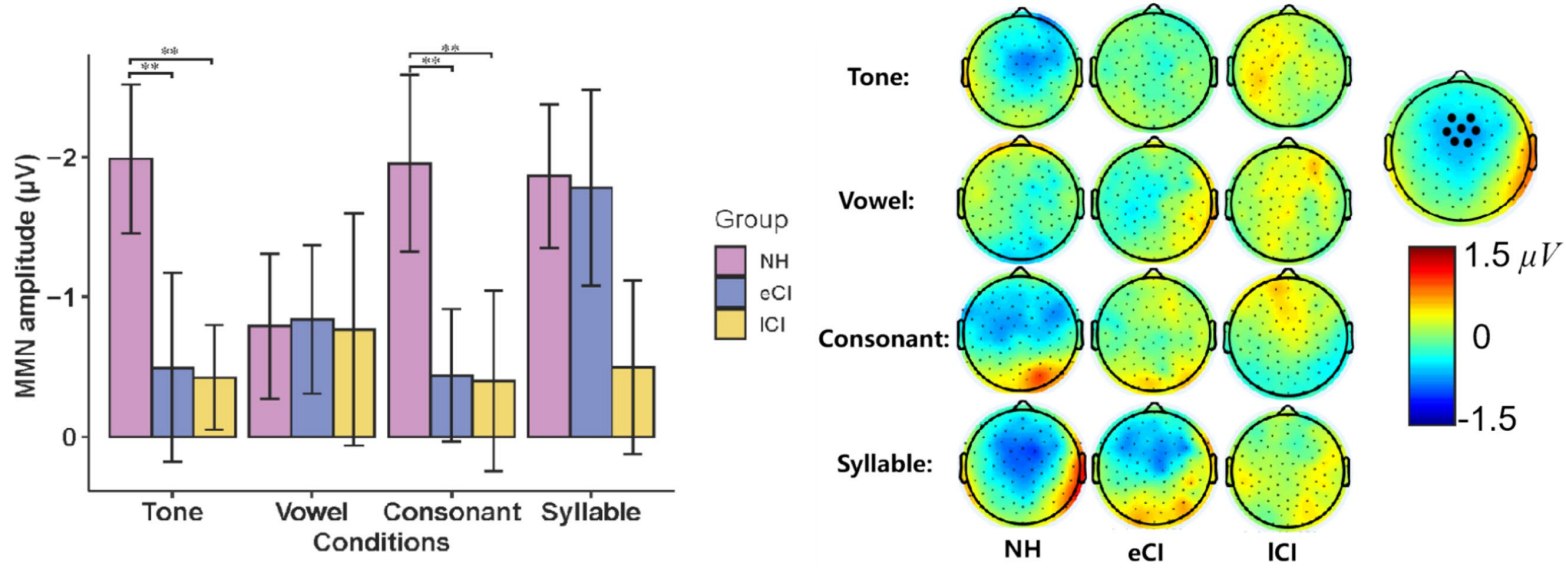
**(Left)** Mismatch negativity (MMN) amplitude during Mandarin Chinese tone condition, vowel condition, consonant condition and syllable condition in children with normal hearing (NH), children with early cochlear implantation (eCI) and children with late cochlear implantation (lCI). **(Right)** The scalp topographies of MMN of each condition and group (*μV*). **p < 0.01 after Bonferroni correction.

The MMN latencies in eCI, lCI and NH children were further analyzed using a two-way ANOVA with condition (tone, vowel, consonant and syllable) as the within-subject factor, and group (eCI, lCI and NH) as the between-subjects factor. For MMN peak latencies, there was no significant effect of condition [*F*_(3,120)_ = 0.808, *p* = 0.491, η_p_^2^ = 0.015], group [*F*_(2, 40)_ = 0.962, *p* = 0.388, *η_p_^2^* = 0.034] nor interaction between these two factors [*F*_(6,162)_ = 0.600, *p* = 0.730, *η*_p_^2^ = 0.022]. Consisted with the waveform analysis, larger MMN amplitudes were found in Tone and Consonant conditions in NH group. Unlike CI group, MMN in NH group had a more fronto-central scalp topography as show in Figures 4, 5.

### 3.4 Larger LDN amplitude for the tone and vowel conditions in lCI children

The LDN waveforms were shown in Figure 4. The results showed a significant interaction between group and condition [*F*_(5.268, 142.226)_ = 3.634, *p* = 0.003, *η_p_^2^* = 0.119]. The results of further post-hoc tests showed that the LDN amplitudes were significantly larger in lCI children than in eCI children in the tone [*p* = 0.043] and in the vowel [*p* = 0.003] conditions, but not in the consonant and syllable conditions. The main effect of the group was noticed to be significant [*F*_(2, 54)_ = 7.057, *p* = 0.002, *η_p_^2^* = 0.207]. The LDN amplitudes of the lCI children were significantly larger than those of the eCI children [*p* = 0.002] and marginally larger than those of the NH children [*p* = 0.066]. A significant effect of condition was also observed [*F*_(2.634, 142.226)_ = 3.013, *p* = 0.038, *η_p_^2^* = 0.053]. LDN amplitudes were significantly smaller in the syllable condition than in the tone [*p* = 0.032] and the consonant [*p* = 0.024] conditions (Figure 5).

**Figure 6:**
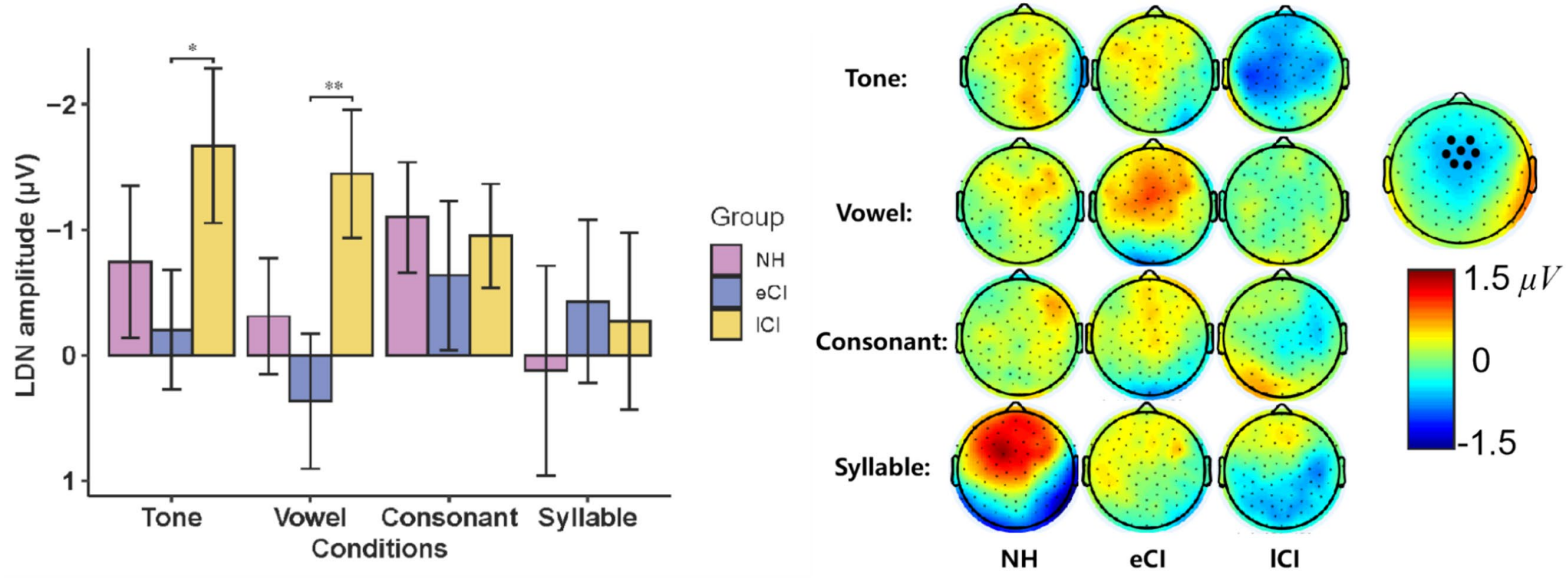
**(Left)** Late discriminative negativity (LDN) amplitude during Mandarin Chinese tone condition, vowel condition, consonant condition and syllable condition in children with normal hearing (NH), children with early cochlear implantation (eCI) and children with late cochlear implantation (lCI). **(Right)** The scalp topographies of LDN of each condition and group (*μV*). *p < 0.05, **p < 0.01 after Bonferroni correction.

For LDN latencies, there was no significant effect of condition [*F*_(3, 162)_ = 0.451, *p* = 0.717, *η_p_^2^* = 0.008], group [*F*_(2, 54)_ = 0.237, *p* = 0.790, *η_p_^2^* = 0.009] or interaction between these two factors [*F*_(6, 162)_ = 1.073, *p* = 0.381, *η_p_^2^* = 0.038]. Consisted with the waveform analysis, larger LDN amplitudes were found in tone and vowel conditions in lCI group, and the lCI group had a more fronto-central scalp topography as show in Figures 4, 6.

Although we included the age as a covariate in the statistical analysis, given the important role of age in language development, we further divided the NH children into two groups (younger group: *n* = 10; older group: *n* = 11) based on the median age to better illustrate that the differences among groups found in the study were due to implantation age rather than other factors. Using the Mann–Whitney non-parametric test, we found no significant differences in non-verbal IQ scores between the younger and older groups [*W* = 59, *p* = 0.073]. We further employed aligned ranks transformation ANOVA, a non-parametric method that allows for multiple independent variables, interactions and repeated measures (Leys and Schumann, 2010). For the behavioral accuracy and reaction time, we did not find a significant main effect of group [accuracy: *F*_(1,19)_ = 1.016, *p* = 0.326, *η_p_^2^* = 0.051; reaction time: *F*_(1,19)_ = 2.313, *p* = 0.145, *η_p_^2^* = 0.109], and the interaction between group and condition [accuracy: *F*_(2,38)_ = 1.507, *p* = 0.234, *η_p_^2^* = 0.074; reaction time: *F*_(2,38)_ = 1.140, *p* = 0.331, *η_p_^2^* = 0.057]. There was no significant difference between the younger and older groups for P2 peak latency [*F*_(1, 19)_ = 1.636, *p* = 0.216, *η_p_^2^* = 0.079], and no significant interaction between group and condition [*F*_(3, 57)_ = 1.649, *p* = 0.188, *η_p_^2^* = 0.080]. There was no significant difference among groups for MMN amplitude, MMN latency, LDN amplitude and LDN latency [MMN amplitude: *F*_(1, 19)_ = 0.0003, *p* = 0.986, *η_p_^2^* = 1.589e-05; MMN latency: *F*_(1, 19)_ = 0.078, *p* = 0.783, *η_p_^2^* = 0.004; LDN amplitude: *F*_(1, 19)_ = 1.099, *p* = 0.308, *η_p_^2^* = 0.055; LDN latency: *F*_(1, 19)_ = 0.364, *p* = 0.554, *η_p_^2^* = 0.018], and no significant interaction between group and condition [MMN amplitude: *F*_(3, 57)_ = 1.857, *p* = 0.147, *η_p_^2^* = 0.089; MMN latency: *F*_(3, 57)_ = 0.608, *p* = 0.613, *η_p_^2^* = 0.031; LDN amplitude _(3, 57)_ = 0.919, *p* = 0.438, *η_p_^2^* = 0.046; LDN latency: *F*_(3, 57)_ = 0.116, *p* = 0.950, *η_p_^2^* = 0.006].

### 3.5 Correlation between behavioral and neural measures and age of CI

**Figure 7:**
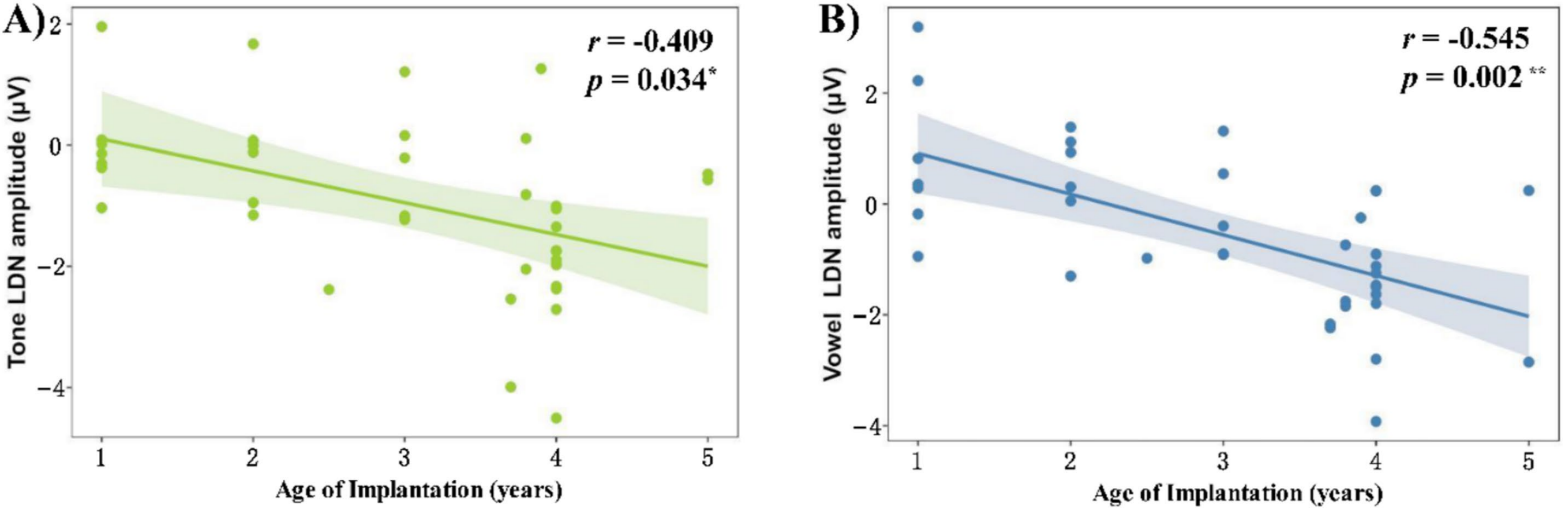
Correlation between age of cochlear implantation and LDN amplitude in the **(A)** tone condition and **(B)** vowel condition.

In addition to dividing children with CI into two groups, we also used age of implantation as a continuous variable, and correlated the behavioral accuracy and ERP amplitudes with the age of implantation as shown in Figure 7.

We found no significant correlation between age of cochlear implantation and accuracy in the tone condition [*r* = −0.239, *p_fdr_* = 0.172], but a trend towards a significant correlation in the vowel condition [*r* = −0.337, *p_fdr_* = 0.092].

We found no significant correlation between age of implantation and MMN amplitude in the tone [*r* = −0.018, *p_fdr_* = 0.925] and consonant [*r* = −0.038, *p_fdr_* = 0.925] conditions, but we found significant correlations between age of implantation and LDN amplitude in the tone [*r* = −0.409, *p_fdr_* = 0.034] and vowel [*r* = −0.545, *p_fdr_* = 0.002] conditions (Figure 7). In other words, the LDN amplitude in CI groups is greater for both tone and vowel conditions when the age at cochlear implantation is older.

All correlations were calculated using Pearson correlation analysis (Benesty et al., 2009) to quantify the linear relationship between variables, followed by False Discovery Rate (FDR) correction using Benjamini-Hochberg method (Benjamini and Hochberg, 1995) to enhance the robustness and reliability of our findings. All correlation and correction operations were applied with Matlab functions.

## 4 Discussion

The present study investigated the effect of implantation age on Mandarin phoneme and syllable perception in CI children with a rehabilitation period of approximately 5 years. Although previous studies have investigated similar issues, most studies have only examined the phoneme and syllable perception abilities of CI children after short-term rehabilitation (i.e., less than 2 years after CI surgery) (Kral and Sharma, 2012; Sharma and Campbell, 2011; Zhou et al., 2013). Since the phonemes that make up Chinese syllables have different sensitive periods in language acquisition (Hua and Dodd, 2000), it is not appropriate to systematically investigate the perception of Chinese phonemes in CI children with a short rehabilitation period.

In behavioral experiments, the eCI group performed better than the lCI group in the tone and vowel discrimination tasks, but there was no significant difference in accuracy between the two CI groups in the consonant discrimination task. The results found in the vowel condition in this study were consistent with previous studies (Yang et al., 2015), but inconsistent with those of previous studies in the tone and consonant conditions (Wu and Yang, 2003; Zhou et al., 2013). We suggest that the inconsistent results may be related to the selection of rehabilitation duration for CI users. Previous studies have tended to select subjects with a rehabilitation duration of about 2 years. Considering the critical period of Mandarin phoneme acquisition, if CI children with a short rehabilitation duration were selected, the age range of CI children may not be within the critical period, making it impossible for us to accurately measure the phoneme perception ability of children with CI.

Previous studies have found that early auditory deprivation may leave the brain, especially the auditory cortex, in a relatively immature state (Li et al., 2012). However, with cochlear implantation, brain plasticity occurs and the auditory cortex may develop to varying degrees (Jiwani et al., 2013). This study found that the P2 latency was significantly longer in the lCI group than in the eCI and NH groups and the amplitude was significantly smaller than CI groups, especially the lCI group as shown in Figure 2. Previous studies have shown that the P2 amplitude and latency were more notably correlated with response time for the more difficult task (Kim et al., 2008). Other studies also have reported the P2 peak amplitudes are associated with.

learning processing of phonemes and neural adaption (Deroche et al., 2023; Tong et al., 2009; Tremblay et al., 2014) where larger amplitudes indicated poor performances in auditory leaning adaption tasks. Consequently, we suggest that the characteristics of P2 component may reflect the relatively immature state of auditory cortex in phoneme perception, which also serves as an indicator for revealing the rehabilitation quality from the early auditory deprivation.

In addition, there were differences in LDN amplitudes between the eCI and lCI groups in the tone and vowel conditions. Two possible explanations for the functions of the LDN component have been proposed. One explanation is that the LDN component may reflect pre-attentive cognitive assessment of acoustic stimuli (both speech and non-speech), and because the latency of the LDN component is longer than that of the MMN component, the LDN component does not reflect sensory processing as the MMN does (Čeponiene et al., 2004). Another view is that the LDN component reflects the influence of other sensory cortices on the auditory cortex (Barry et al., 2009; Hill et al., 2004). Indeed, both explanations suggest that the LDN component may be involved in the interaction between the auditory cortex and other sensory cortices. In particular, auditory deprivation and postoperative auditory reconstruction may cause the brain plasticity. We found differences in tone and vowel condition between eCI and lCI groups, which may be because lCI cannot grasp tone and vowel well after missing the critical period for tone and vowel acquisition. That is the reason why we found larger LDN component in lCI children. Therefore, we speculate the LDN component may be closely related to the brain plasticity. A study used pre-operative brain morphological data from cochlear implantation patients before 3.5 years of age to predict postoperative speech perception. It was found that brain regions unaffected by auditory deprivation played an important role in postoperative speech ability (Feng et al., 2018). At the same time, these higher brain regions can compensate for processing deficits due to the immature development of the primary auditory cortex, and can modulate the primary auditory cortex in a top-down feedback manner. In the lCI group, the longer P2 latency and larger amplitude indicated the underdevelopment of the primary auditory cortex due to auditory deprivation. At this point, the higher cortex modulates the primary auditory cortex, resulting in larger LDN amplitudes in lCI group. As the critical period for the acquisition of tone and vowel occur early in life, the larger LDN amplitudes of the lCI group was mainly reflect in tone and vowel conditions.

In the tone and consonant conditions, we found that the MMN amplitude of the NH group was significantly larger than that of the eCI and lCI groups, but there was no difference in the MMN component between the two CI groups. These results were inconsistent with recent findings by Hu et al. (2021), who compared the brain activity of CI and NH groups during tone perception and found that the CI and NH groups had similar patterns of the MMN component. We speculate that this may be related to subject selection (Hu et al., 2021). Some participants in the Hu et al. study had bilateral implants. Studies have found that children with bilateral implants have better speech perception than children with unilateral implant in quiet or noisy environments (Litovsky et al., 2006; Sparreboom et al., 2010). However, all subjects in our study had unilateral implant. Future studies are needed to investigate the differences in speech processing between children with unilateral implant and children with bilateral implants.

MMN is primarily generated in the tonotopically organized primary auditory cortex. It requires auditory stimulation in uteri from about gestation week 21. There are also critical developmental windows after which the neural organization remains impaired (Chang and Kanold, 2021). The lack of MMN generation in CI may indicate a fundamental cortical hearing deficit which may be partially corrected via alternative pathways. The differential results observed in this study concerning MMN between CI and NH Groups support the notion that there is another critical period for the development of the primary auditory cortex in hearing-impaired children, which occurs from birth to approximately 3.5 years of age (Huttenlocher and Dabholkar, 1997; Sharma et al., 2015). Identifying and capitalizing on this critical period for cochlear implantation and subsequent training may yield significant positive outcomes in terms of auditory development.

MMN and LDN components may jointly influence phoneme perception in CI children. Although previous studies have found the MMN component to be an important indicator of speech perception ability, we only found differences in MMN amplitude between the NH and CI groups, and no difference in MMN amplitudes between two CI groups. Interestingly, the difference between the eCI and lCI groups was mainly in the amplitudes of the LDN component, which was associated with modulation from higher cortex to primary auditory cortex. Furthermore, the lCI group performed worse than the eCI group on the tone and vowel discrimination. It is worth noting that the amplitude of the LDN component in the tone and vowel conditions was larger when the age of cochlear implantation is older, suggesting that the LDN component may reflect the difference in phoneme perception between two groups due to the age of implantation. Taken together with the above findings, we suggest that auditory discrimination may reflect the interaction of MMN and LDN components. Specifically, the NH group performed better than the two CI groups in the tone discrimination task, as reflected in the larger amplitude of the MMN components in the NH group; the difference between the eCI and lCI groups was mainly reflected in the LDN component, the behavioral performance of the two CI groups may be result in the combined effect of insufficient early sensory processing of the auditory cortex and compensation of additional cognitive processing later. A similar result appeared in the vowel condition, i.e., the difference in the LDN amplitudes between the eCI and lCI groups, which was consistent with the finding that the lCI group performed worse than the eCI group. In future studies we will discuss how these two components work together to influence behavioral performance.

There are still some shortcomings in this study. First, although the results of this study showed that the age of implantation had an effect on the Chinese phoneme perception of CI children, it could not answer the question of how the phoneme perception of CI children changed with increasing rehabilitation duration. Therefore, future studies need to conduct a dynamic analysis of pre- and post-operative phoneme perception in CI children at different implantation ages. In addition, this study mainly investigated the phoneme perception of CI children. In fact, word and sentence processing abilities are also important components in the assessment of CI children’s language skills. Therefore, future research should also consider the effect of implantation age on high-level language skills. We also encounter two substantial obstacles that have precluded us from accurately recording the precise dates of cochlear implantation (CI). First, since these children had their cochlear implants for more than 5 years before the EEG data was collected, their parents could not remember the exact surgery date accurately. Second, many of the children who underwent EEG had their implants done at other hospitals, making it hard for us to find out the exact dates. Consequently, greater efforts are warranted in our future research endeavors to ensure the acquisition of more precise age data.

## 5 Conclusion

This study found that children with late implantation had weaker tone and vowel perception indicated by MMN and LDN components using a more suitable paradigm. The MMN and LDN components together reflect the delay in tone and vowel perception in children with late implantation. Consistent with the results of the behavioral tests, the differences of P2 component latency and amplitude may reflect the abilities in phoneme perception between children in the NH and CI groups. The MMN component might be an indicator to distinguish the perceptual abilities of the NH and CI groups. The LDN component may be the indicator of Chinese phonemes in children with cochlear implant.

## Data Availability

The raw data supporting the conclusions of this article will be made available by the authors, without undue reservation.
