# Supplementary material for: Tone and vowel perception delay: long-term effects of late cochlear implant in children with prelingual deafness
Source: Front Hum Neurosci. 2025 Mar 11;19:1516931. doi: 10.3389/fnhum.2025.1516931 (PMC11933052; doi:10.3389/fnhum.2025.1516931)
Supplement: Supplementary file 1 [file Data_Sheet_1.pdf]

Appendices

Table 1

Demographics data of children with early cochlear implantation and late cochlear implantation.

ID	Age	Gender	Age of Implantation	Implant use	Implanted ear	CI type
EC1	6.44	F	1	5.44	R	MED-EL
EC2	5.84	M	1	4.84	R	MED-EL
EC3	8.07	F	3	5.07	R	MED-EL
EC4	12.49	M	2	10.49	R	MED-EL
EC5	6.87	M	2	4.87	R	MED-EL
EC6	11.29	M	3	8.29	R	MED-EL
EC7	6.08	F	1	5.08	R	MED-EL
EC8	10.65	F	2	8.65	R	MED-EL
EC9	6.5	F	1	5.5	R	MED-EL
EC10	7.43	M	3	4.43	R	MED-EL
EC11	8	M	2	6	R	MED-EL
EC12	8	M	2	6	R	MED-EL
EC13	8.82	F	3	5.82	R	MED-EL
EC14	12.44	F	3	9.44	R	MED-EL
EC15	7.43	F	1	6.43	R	MED-EL
EC16	8.2	M	1	7.2	R	MED-EL
EC17	6.41	F	2	4.41	R	MED-EL
EC18	8.03	M	2.5	5.53	R	MED-EL
EC19	6.02	M	1	5.02	R	MED-EL
LC1	9	F	3.9	5.1	R	MED-EL
LC2	12	M	5	7	R	MED-EL
LC3	9.73	M	4	5.73	R	MED-EL
LC4	8.42	F	4	4.42	R	MED-EL
LC5	8.925	M	4	4.925	R	MED-EL
LC6	10.02	F	4	6.2	R	MED-EL

LC7	8.42	M	3.7	4.72	R	MED-EL
LC8	10.97	M	3.7	7.27	R	MED-EL
LC9	8.45	M	3.8	4.65	R	MED-EL
LC10	8.8	M	4	4.8	R	MED-EL
LC11	6.5	F	3.8	4.8	R	MED-EL
LC12	7.17	M	4	3.17	R	MED-EL
LC13	11.15	F	4	7.15	R	MED-EL
LC14	10.3	F	5	5.3	R	MED-EL
LC15	10.65	F	4	6.65	R	MED-EL
LC16	10	F	4	6	R	MED-EL
LC17	8.72	F	4	4.72	R	MED-EL
LC18	8.16	F	3.8	4.36	R	MED-EL
LC19	11.25	M	4	7.25	R	MED-EL
